# Comparative transcriptomic analysis of two important life stages of *Angiostrongylus cantonensis*: fifth-stage larvae and female adults

**DOI:** 10.1590/1678-4685-GMB-2016-0274

**Published:** 2017

**Authors:** Liang Yu, Binbin Cao, Ying Long, Meks Tukayo, Chonglv Feng, Wenzhen Fang, Damin Luo

**Affiliations:** 1 Department of Biology, School of Life Sciences, Xiamen University, Xiamen, Fujian, 361102, China; 2 State Key Laboratory of Cellular Stress Biology, Xiamen University, Xiamen, Fujian, 361102, China; 3 Translational Medicine Center, Hunan Cancer Hospital, Changsha, Hunan, 410006, China; 4 College of the Environment & Ecology, Xiamen University, Xiamen, Fujian, 361102, China

**Keywords:** comparative transcriptomes, cuticle synthesis, lysine degradation, lysosomal pathway, metallopeptidases

## Abstract

The mechanisms involved in the fast growth of *Angiostrongylus cantonensis* from fifth-stage larvae (L5) to female adults and how L5 breaks through the blood-brain barrier in a permissive host remain unclear. In this work, we compared the transcriptomes of these two life stages to identify the main factors involved in the rapid growth and transition to adulthood. RNA samples from the two stages were sequenced and assembled *de novo*. Gene Ontology and Kyoto Encyclopedia of Genes and Genomes pathway analyses of 1,346 differentially expressed genes between L5 and female adults was then undertaken. Based on a combination of analytical results and developmental characteristics, we suggest that *A. cantonensis* synthesizes a large amount of cuticle in L5 to allow body dilatation in the rapid growth period. Products that are degraded via the lysosomal pathway may provide sufficient raw materials for cuticle production. In addition, metallopeptidases may play a key role in parasite penetration of the blood-brain barrier during migration from the brain. Overall, these results indicate that the profiles of each transcriptome are tailored to the need for survival in each developmental stage.

## Introduction

*Angiostrongylus cantonensis*, a parasitic nematode (roundworm) that causes angiostrongyliasis, has been detected in lemurs, opossums, tamarins, falcons and non-human primates, and may endanger many wildlife species in areas where the infection is uncontrolled (Martins *et al.*, 2015; Spratt, 2015). In addition, since the first clinical diagnosis reported in Taiwan in 1945, the disease has been increasingly observed worldwide, including in previously non-epidemic countries such as France, Germany, the Caribbean region (including Jamaica), Brazil, Ecuador and South Africa (Morassutti *et al.*, 2013; Barratt *et al.*, 2016). Angiostrongyliasis has been considered as an emerging global public health problem mainly because of the increase of international traffic facility which make it endemic previously in many unaffected areas (Graeff-Teixeira, 2007).

*Angiostrongylus cantonensis* has a complicated life cycle. The first-stage larvae (L1) develop to third-stage larvae (L3) in about two weeks in intermediate hosts such as snails and slugs. When intermediate hosts with L3 are swallowed by permissive hosts, which are usually rats, the parasites finally develop to adults and live in the hosts pulmonary arteries and heart (Long *et al.*, 2015). However, if positive snails are eaten by non-permissive hosts such as humans and mice, the course of infection usually terminates at L5 in the host brain. When the parasites develop from L5 to adult in permissive hosts, the most notable physical change during this period is expansion of the magniloquent body size and this is generally considered to be the parasites most vigorous period of growth (Hu *et al.*, 2012; Wang *et al.*, 2015). Accordingly, energy and material metabolism are greater during this period than in any other life stages. At the same, there is more intense parasite-host interaction that is mediated by greater release of parasite excretion/secretion protein (ESP) and greater uptake of material from the host.

To reach its final parasitic sites, L5 needs to break through the blood-brain barrier when moving out of the brain (Chiu and Lai, 2014). Proteolytic enzymes are assumed to be involved in blood-brain barrier disruption. A comprehensive understanding of the special developmental processes requires a comparison of the L5 and adult life stages to identify key factors related to rapid growth, parasite-host interaction and transmigration. In this regard, next generation sequencing (NGS), an increasingly popular and effective technology for genome-wide analysis of transcript sequences (t’ Hoen *et al.*, 2013), has been successfully applied to transcriptomic profiling and characterization in the Strongylida and many other parasitic helminths (Chilton *et al.*, 2006).

In this study, the transcriptomes of L5 and female adults of *A. cantonenesis* were sequenced by NGS and assembled *de novo* with Trinity. Subsequently, genes that were differentially expressed between these two life-stages were identified by comparative transcriptomic analysis. Quantitative real-time polymerase chain reaction (qPCR) was used to validate the transcriptomic data. The findings described here provide a general picture of the gene expression profiles of *A. cantonensis* that may shed light on the development of this parasite and its survival in the host.

## Material and Methods

### Animals

Female Sprague Dawley (SD) rats (6-8 week-old, 120-150 g) and *Pomacea canaliculata* (channeled applesnail) were used to maintain the parasites and allow completion of the whole life cycle. The rats were housed with free access to water and food in the Xiamen University Laboratory Animal Center. This study was done in strict accordance with the Regulations for the Administration of Affairs Concerning Experimental Animals (as approved by the State Council of the People's Republic of China). Moreover, the protocols involving rats were approved by the Committee for the Care and Ethics of Xiamen University Laboratory Animals (permit no. XMULAC2012-0122). L5 were obtained from the brains of rats infected with 200 L3 15 days previously. Female adult worms were collected as previously described (Zhang *et al.*, 2014). The worms were washed three times with phosphate-buffered saline (PBS; 137 mM NaCl, 2.7 mM KCl, 10 mM Na_2_HPO_4_, 1.8 mM KH_2_PO_4_, pH 7.4) and then stored a −80 °C after soaking in RNAhold reagent (Transgene Biotech, Beijing, China) until RNA extraction.

### RNA extraction, cDNA library preparation and RNA sequencing

Total RNA from each life stage was extracted with TransZol Up (Transgene Biotech) according to the manufacturer's protocols. The RNA concentration was measured using a Qubit^®^ RNA assay kit in a Qubit^®^ 2.0 Fluorometer (Life Technologies, Carlsbad, CA, USA). Two sequencing libraries were generated and 125 bp pair end-sequencing was done on an Illumina HiSeq 2500 platform by Novogene Bioinformatics Technology Co., Ltd. (Beijing, China).

### Data quality control and transcriptome assembly

Raw data (raw reads) in fastq format were first processed through in-house perl scripts to obtain clean data (clean reads) by discarding reads containing adapters, reads containing ploy-N and low quality reads. Parent transcriptome (P transcriptome) assembly was done based on clean data from two samples using Trinity software (v.2.0.6), with all parameters set as default (Grabherr *et al.*, 2011). Before annotation, unigenes were picked from the P transcriptome with CD-hit (Li and Godzik, 2006). Intactness of the assembled P transcriptome was assessed with the software tool BUSCO (Benchmarking Universal Single-Copy Orthologs) that is based on evolutionarily informed expectations of gene content, with default settings (Simão *et al.*, 2015). The unigenes were then annotated with BLASTx (BLAST + v.2.2.25) by querying these to the following databases: NCBI non-redundant protein sequences (Nr), NCBI non-redundant nucleotide sequences (Nt), the Protein Family database (Pfam), Swiss-Prot, Gene Ontology (GO), the Eukaryotic Orthologous Groups database (KOG) and the Kyoto Encyclopedia of Genes and Genomes (KEGG). The E-value cutoff was set as 1 × 10^−5^.

### Gene expression quantification and differentially expressed genes (DEGs) screening

The transcriptomes of L5 and female adults were assembled with the clean data and the P transcriptome was set as the reference. Gene expression levels were estimated by RSEM (v.1.2.15) for each sample, as described (Li and Dewey, 2011). First, the read count for each gene was obtained from the result of clean data mapped back onto the assembled P transcriptome. Subsequently, the read count of each sequenced library was adjusted with the edger program package to ‘reads per kilobase per million mapped’ (RPKM). The differential expression analysis of two samples was done using the DEGseq R package (v.1.12.0) (Wang *et al.*, 2010). The P-value was adjusted by the q-value; a q-value < 0.005 and |log_2_ (fold change)| > 1 were set as the threshold criteria for significant differential expression.

### GO and KEGG pathway enrichment analysis of DEGs

For functional annotation, GO enrichment analysis of DEGs was implemented using the GOseq R packages (v1.10.0) based on a Wallenius non-central hyper-geometric distribution (Young *et al.*, 2010). KEGG pathway analysis of DEGs was then done through the KEGG database (http://www.genome.jp/kegg/) (Mao *et al.*, 2005; Kanehisa *et al.*, 2008).

### cDNA synthesis and quantitative real-time PCR (qPCR)

One microgram of RNA from each stage was converted to first strand cDNA using a First Strand cDNA synthesis kit (TaKaRa, Dalian, China), according to the manufacturer's instructions. Fifteen DEGs were significantly enriched in the lysosomal pathway and were randomly chosen for validation by qPCR. β-Actin was selected as an internal control based on previous studies (Zhang *et al.*, 2014; Long *et al.*, 2016). Primers were designed using Primer 3.0 and the sequences are listed in the supplementary materials (Table S1). Three biological replicates were used for the qPCRs of each gene. The reaction mixture (10 μL) consisted of 5 μL of SYBR Green PCR master mix, 0.5 μL (10 μM) of the forward and reverse primers, 1 μL of cDNA (diluted 10 times with double distilled water – ddH_2_O) from each developmental stage and 3 μL of ddH_2_O. The cycling conditions involved an initial activation step at 95 °C for 30 s, followed by 40 cycles of 95 °C for 5 s, 60 °C for 30 s and fluorescence acquisition at 60 °C for 30 s using a CFX96 Real Time system (BioRad, USA).

### Statistical methods

Statistical analysis of the qPCR data was done with SPSS18.0 (SPSS statistical package for Windows^®^) and Students *t*-test was used to detect significant differences. A p-value < 0.05 indicated significance.

## Results

### Summary of the raw sequence reads and assembly

To obtain a global overview of the *A. cantonensis* transcriptome, clean data from each life stage were combined to provide a relatively comprehensive gene pool. Overall, 12.66 Gb of clean data (~50-fold coverage of the whole genome) were used for *de novo* transcriptome assembly. Raw reads of the transcriptome have been deposited in the NCBI Short Read Archive (SRA, http://www.ncbi.nlm.nih.gov/sra/) under accession numbers SRR3199277 and SRR3199278. Table 1 summarizes the sequence reads and P transcriptome. BUSCO analysis revealed that the P transcriptome was largely complete as we recovered 531 complete single-copy BUSCOs (68.1%) and an additional 71 fragmented BUSCOs (8.4%). Only 5.1% of the BUSCOs were found to be duplicated in the combined transcriptome, indicating that the transcriptome assembly was successful.

**Table 1:** Summary of the sequence reads and P transcriptome.

Summary of sequence reads		
Sample	F	L5
Raw reads	55,725,337	50,234,050
Clean reads	51,957,988	49,275,894
Clean reads (%)	93.24	98.09
Clean bases (Gb)	6.5	6.16
Q20 of clean reads (%)	96.09	96.89
Total clean bases (Gb)	12.66	
Characteristics of the P transcriptome		
Levels	Transcripts	Unigenes
Total nucleotides	99,597,159	51,401,554
Numbers (length > 200 bp) (n)	115,369	82,769
Average length (bp)	863	621
N50	1,731	949
Shortest transcript (bp)	201	
Longest transcript (bp)	20,809	

### Annotation of the P transcriptome

**Figure 1:**
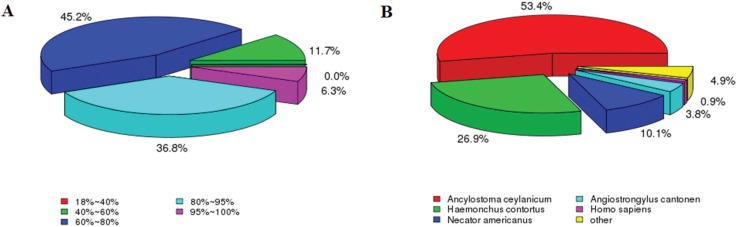
Summary of the results of sequence-homology searches against the Nr database. (A) Similarity distribution of the closest BLASTX matches for each sequence. (B) A species-based distribution of BLASTX matches for sequences.

Most (98.69%) of the P transcriptome unigenes were successfully annotated in less than one of the seven public databases indicated above. The homology and species-based distribution for all of proteins were analyzed against the Nr database and 82% of the sequences showed similarity > 60% with their blast result. *Ancylostoma ceylanicum, Haemonchus contortus* and *Necator americanus* showed the greatest similarity in the species-based distribution (Figure 1).

**Figure 2:**
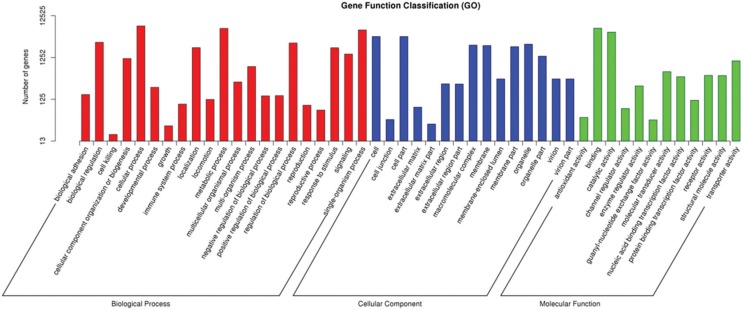
Gene ontology classifications of unigenes from the P transcriptome.

GO analysis assigned 12,525 unigenes to the corresponding GO terms, the details of which are shown in Figure 2. Most of the biological process (BP) categories were related to cellular processes (GO: 0009987, 56.73%), metabolic processes (GO: 0008152, 49.64%) and single-organism processes (GO: 0044699, 45.54%), while most unigenes were sorted into the cell part (GO: 0044464, 31.67%), cell (GO: 0005623, 31.67%) and organelle (GO: 0043226, 20.77%) in the cell component (CC) category. Binding (GO: 0005488, 50.05%) and catalytic activity (GO: 0003824, 40.3%) were the main GO terms of the molecular function (MF) category.

**Figure 3:**
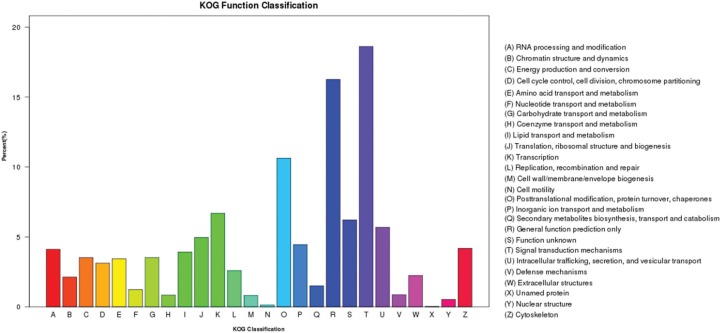
KOG classifications of unigenes from the P transcriptome.

Blasting to the KOG database for functional prediction and classification assigned 6,251 unigenes to 26 specific pathways (Figure 3). ‘Signal transduction mechanisms’ (1,163) was the largest group, followed by ‘General function prediction only’ (1,016), ‘Post-translational modification, protein turnover, chaperones’ (664), and ‘Transcription’ (418).

**Figure 4:**
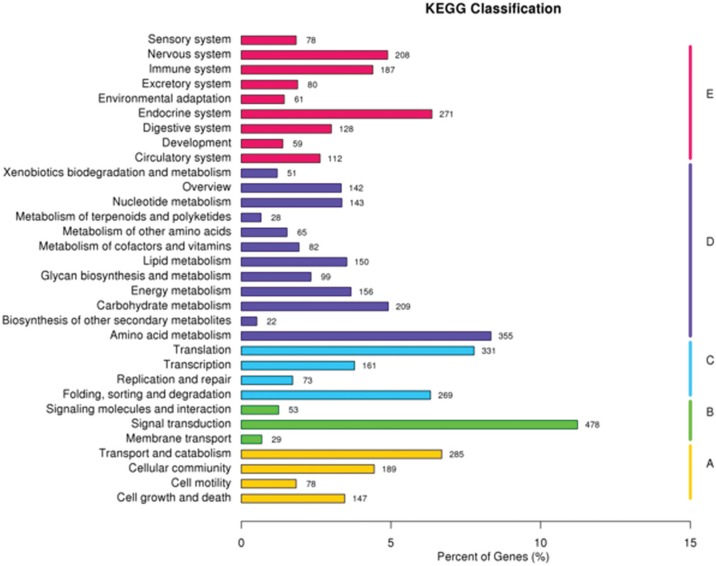
KEGG classifications of unigenes from the P transcriptome. (A) Cellular processes, (B) Environmental information processing, (C) Genetic information processing, (D) Metabolism and (E) Organismal systems.

A total of 4,257 unigenes were functionally assigned to the 259 KEGG pathways of five KEGG categories by KEGG pathway analysis. Lysine degradation (ko00310), purine metabolism (ko00230) and ribosome (ko03010) were the top three pathways sorted by the unigenes numbers involved (Table S2). The distribution of these unigenes in 32 KEGG sub-categories is shown in Figure 4. Endocrine system, amino acid metabolism, translation, signal transduction, transport and catabolism were the highest enriched subcategories of each category.

### Gene expression quantification and related analyses

**Figure 5:**
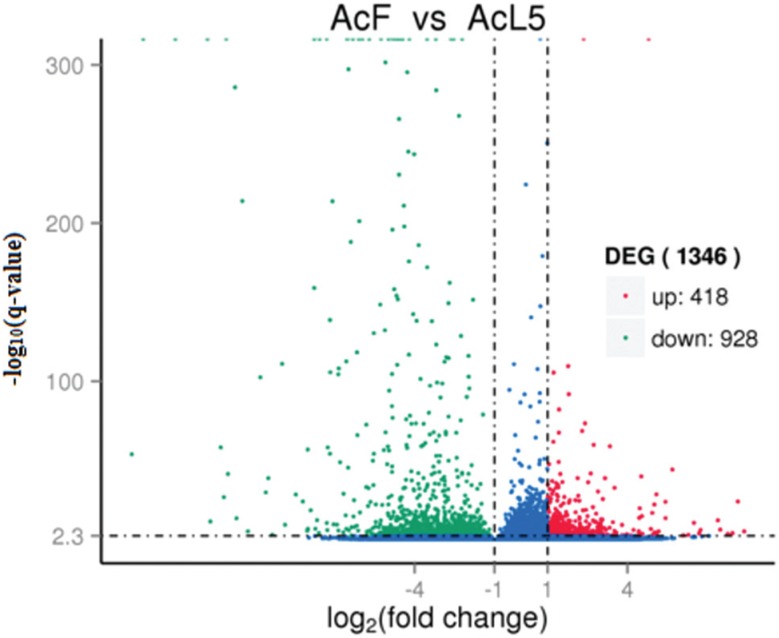
Volcano plots of differentially expressed genes (DEGs) in L5 and female adults. AcF – *A. cantonensis* female, AcL5 – young adults of *A. cantonensis*. Unigenes that satisfied these conditions (log_2_-fold-change > 1 and q value < 0.005) were considered to be differentially expressed. Blue, red and green splashes represent genes with no significant change in expression, significantly up-regulated genes and significantly down-regulated genes, respectively.

**Figure 6:**
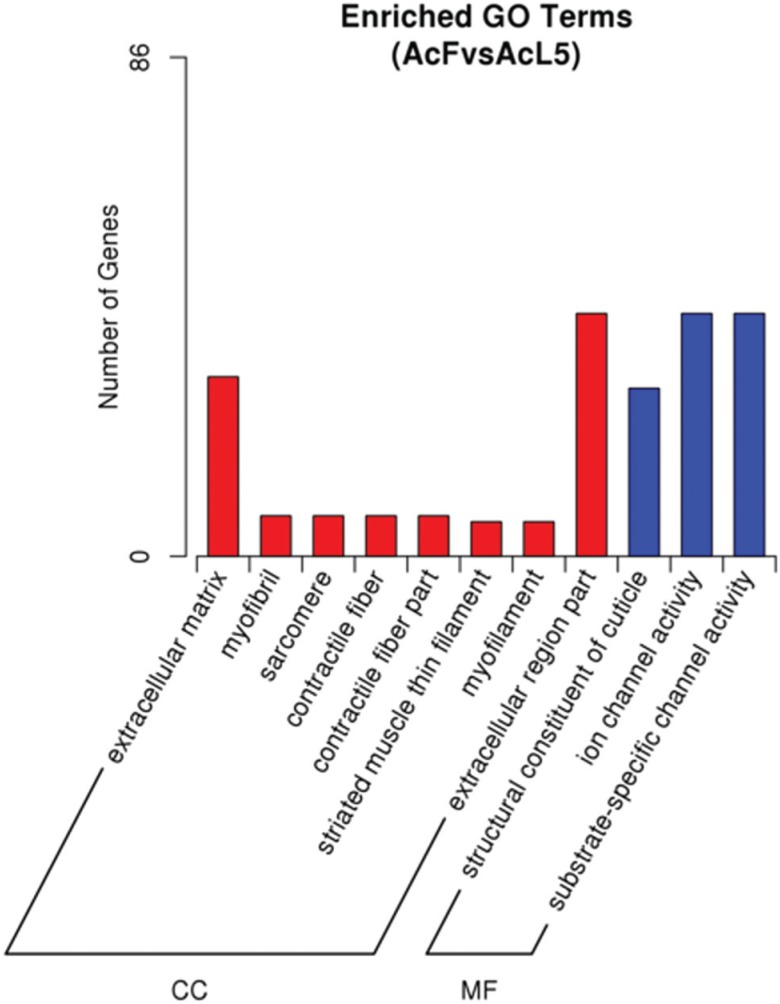
GO enrichment analysis results for down-regulated DEGs. CC – cellular component and MF – molecular function.

Gene expression quantification was done as described in the Methods section. The mapping rate for L5 and female adults was 87.15 and 82.94, respectively. The percentages of both samples were > 80%, suggesting that the transcriptome was well-assembled. Of the 1,346 DEGs analyzed (L5 *vs* female adults), 418 were up-regulated and 928 down-regulated (Figure 5). c6135_g1 was the highest up-regulated gene with a log_2_ fold-change of 8.15; annotation information suggested that this gene was involved in nematode cuticle collagen synthesis. GO enrichment analysis of 1,346 DEGs showed that the extracellular region part (GO: 0044421), ion channel activity (GO: 0005216) and substrate-specific channel activity (GO: 0022838) were the top three of the 11 significantly enriched terms (Figure 6).

**Figure 7:**
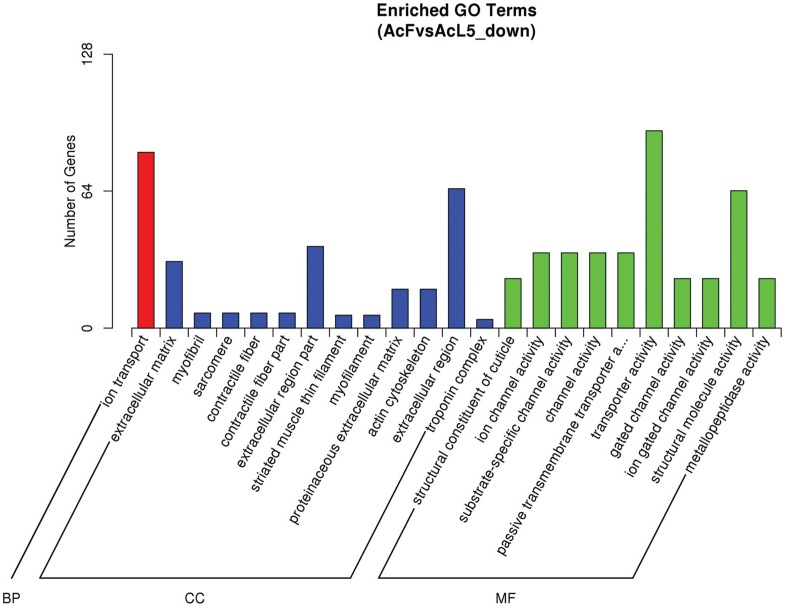
GO enrichment analysis results for down-regulated DEGs. BP – biological process, CC – cellular component and MF – molecular function.

Nine hundred and twenty-eight down-regulated unigenes were analyzed in the same way and revealed 23 significantly enriched GO terms (Figure 7). Transporter activity and structural molecule activity were the dominant enriched terms of the MF category. Ion transport was the sole term in the BP category, which had 82 unigenes. In the CC category, the extracellular region, extracellular region part and extracellular matrix were the most highly enriched.

**Figure 8:**
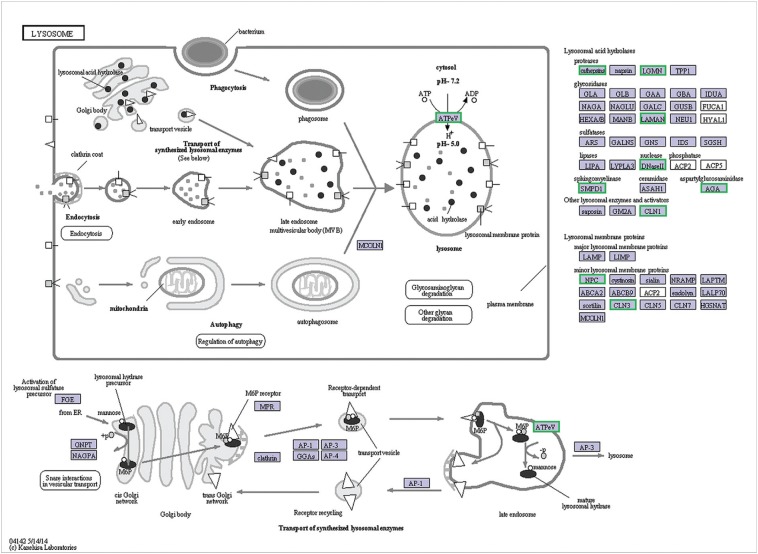
Unigenes predicted to be involved in the lysosomal pathway. Green represents down-regulated unigenes in female adults compared to L5.

DEGs were further subjected to the KEGG database for pathway enrichment analysis. Lysosome was the only highly enriched KEGG pathway (Figure 8). Lysosomes are membrane-delimited organelles that serve as the cell's main digestive compartment where various macromolecules are delivered for degradation and recycling. Lysosomes contain > 60 hydrolases involved in this degradation in an acidic environment (~pH 5). Seven of these (legumain [LGMN], cathepsins, N4-[β-N-acetylglucosaminyl]-L-asparaginase [AGA], palmitoyl-protein thioesterase, deoxyribonuclease II [DNase II], lysosomal α-mannosidase [LAMAN] and sphingomyelin phosphodiesterase [SMPD1]) were highly expressed in L5. V-type H^+^-transporting ATPase (V-ATPase), battenin (encoded by the CLN3 gene) and Niemann-pick type C (NPC) were the other three proteins associated with the lysosomal membrane and were down-regulated in female adults. The perturbation of these proteins usually leads to marked changes in lysosomal function.

### Validation of DEGs by qPCR

**Figure 9:**
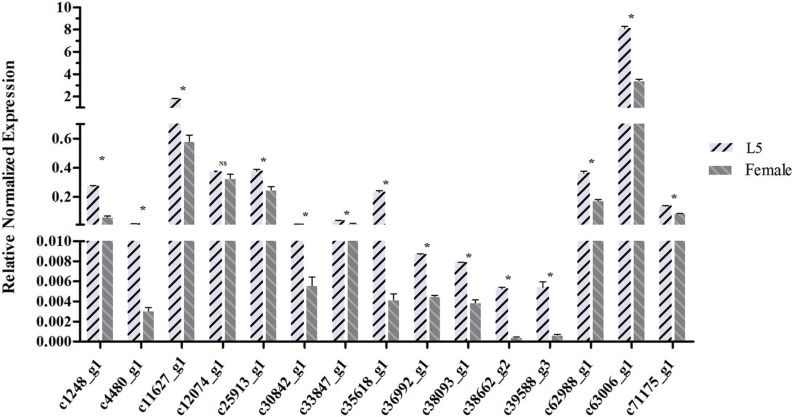
qPCR validations for 15 DEGs in the lysosomal pathway. An asterisk indicates a significant difference between L5 and female adult expression levels (p < 0.05, Student's *t*-test). NS – no significant difference. The columns are the mean ± SEM (n = 3 per column).

Although biological repeats for the RNA-seq data were not done, qPCR (triplicate replicates) was used to check 15 out of 18 DEGs involved in the lysosomal pathway; 14 of the 15 unigenes revealed consistent expression patterns with the RNA-Seq data (Figure 9).

## Discussion

The highest growth rate in *A. cantonensis* occurs from the L5 to the female adult stage, during which period body size increases sharply. In permissive hosts, L5 migrates from the brain to the pulmonary artery via the blood-brain barrier. However, the key factors in this important biological process remain largely unknown. To increase our understanding of this phenomenon, we compared the P transcriptome assembled with clean data obtained from the transcriptomes of L5 and female adults, two important life stages of *A. cantonensis*.

Although two genome assemblies have been reported for *A. cantonensis* (Morassutti *et al.*, 2013; Yong *et al.*, 2015), we did not use them as references for the following reasons. First, the assembly level for the first of these two studies was ‘Conting’ and therefore did not satisfy the requirement for a reference genome. The other study reached the ‘Scaffold’ level, but the Scaffold N50 (43,900) was too small, indicating that the genome was not well assembled. Second, the primary assembly unit did not have any assembled chromosomes or linkage groups so that no effective information could be extracted for downstream analysis. Third, before transcriptome assembly, the clean data for L5 and female adults had been separately mapped onto the genome sequences with Tophat2 (mismatch = 2) and the mapping rates were only 82.5% and 79.4%, respectively (Kim *et al.*, 2013). Fourth, many reports have proven that *de novo* transcriptome assembly can also provide satisfactory results (Fang *et al.*, 2016; Hasanuzzaman *et al.*, 2016; Santos *et al.*, 2016; Wei *et al.*, 2016).

Although Lian and Wang reported that *A. cantonensis* had the highest homology to *Caenorhabditis* spp. (up to 97.87%) (Morassutti *et al.*, 2013; Wang *et al.*, 2013), our Nr annotation species-based distribution revealed that *A. cantonensis* was most similar to *Ancylostoma ceylanicum* (53.4%), followed by *Haemonchus contortus* (26.9%) and *Necator americanus* (10.1%) (Figure 1B). These differences may have been caused by bias in the sequence information of different species stored in public databases. Furthermore, the rapid development of high-throughput sequencing technologies has narrowed the gap between model and non-model species because more information could be retrieved from public databases, even for non-model species such as *A. ceylanicum, H. contortus* and *N. americanus* (Abubucker *et al.*, 2008; Schwarz *et al.*, 2013; Tang *et al.*, 2014). On the other hand, since these three species and *A. cantonensis* are parasitic nematodes, it would be more reasonable for *A. cantonensis* to have a higher homology with these species than with free-living nematodes.

Lysine, an essential amino acid that animals must obtain from their diet because they cannot synthesize it, is not only an important nutritional requirement for growth, but can also directly or indirectly regulate the immune system (Theis *et al.*, 1969; Williams *et al.*, 1997). Lysine-deficient diets result in greater body length in *Ascaridia galli* and a higher worm burden in chickens. A deficiency in dietary lysine limits protein synthesis and compromises antibody responses and cell-mediated immunity in chickens (Chen *et al.*, 2003; Li *et al.*, 2007; Das *et al.*, 2010). Consequently, these parasites show greater growth and development in hosts with impaired immune systems. Various unigenes were found to be involved in the lysine degradation pathway (ko00310, 225) in the P transcriptome, indicating active lysine degradation in these life stages. The consumption of a large amount of host lysine or lysine-containing proteins by these parasites could make the host lysine-deficient, thereby indirectly weakening the host immune system. We therefore speculate that lysine insufficiency resulting from lysine degradation may be a neglected strategy of *A. cantonensis* for escaping immunological detection by the host.

The 23 GO terms and one KEGG pathway were significantly enriched with 928 down-regulated DEGs. Among these, eight of 23 enriched GO terms were related to ion channel transport that is essential for normal cell function (Desai, 2012). When L5 migrate from the brain to the final parasitic site there are significant changes in the physicochemical properties of the external environment of *A. cantonensis*. The concentrations of Na^+^ and Mg^2+^ are higher in cerebrospinal fluid (CSF), whereas the concentrations of K^+^, Ga^2+^, Cl^–^ and HCO_3_^–^ are higher in blood. In addition, the protein and glucose content in CSF is lower than in blood (Pardridge, 2005). Obviously, the parasite must adapt to different habitats by modulating the transmembrane transport of ions and organic solutes. Based on the results of the bioinformatics analysis, the bioprocess associated with ion channels and transporters will presumably be active in this period.

The cuticle or outer surface of all parasitic nematodes has important roles in locomotion (by providing points of attachment for muscle), growth, osmoregulation and parasite-host interactions (Wright, 1987). Cuticles often differ in surface protein expression and composition during the developmental stages in parasitic nematodes. Presumably, the worms adjust to changing developmental needs and environmental conditions, including escape from the hosts immunological system, particularly since cuticles are the target of a variety of host immunological responses that may kill the worms (Davies and Curtis, 2011). The protective collagenous cuticle of these parasites is required for survival and the metalloproteinase astacin is a key enzyme involved in the collagen synthesis pathway. GO analyses of 928 down-regulated DEGs identified many GO terms related to muscles and cuticle formation. Based on this finding, we suggest that *A. cantonensis* invests considerable effort on cuticle and muscle biosynthesis in L5 to satisfy the rapid body expansion in the transition to female adulthood and to survive in different host tissues.

A further 23 enriched DEGs with metallopeptidase activity also attracted our attention. In addition to being associated with collagen synthesis, the essential role of these proteases in tissue invasion is well-documented. The metallopeptidases of bacteria such as *Candida albicans* and *Pseudomonas aeruginosa* are located in the cell wall and can degrade host extracellular matrix, thereby accelerating bacterial invasion (Rodier *et al.*, 1999; Miyoshi and Shinoda, 2000). Similar host penetration functions for related proteases have been detected in many parasitic nematodes, including *Ancylostoma caninum, Necatora mericanus* and *Nippostrongylus brasiliensis* (Hotez *et al.*, 1990; Knox, 2011; Kumar and Pritchard, 1992; Williamson *et al.*, 2003). Moreover, the work of Miyoshi indicated that metalloproteases present in the ESP of *A. cantonensis* infective larvae may suppress the host's immune response and allow parasite migration to the host's central nervous system by degrading human matrix metallopeptidase 9 (MMP-9) (Adisakwattana *et al.*, 2012). Based on these considerations, we speculate that the increased expression of unigenes related to metallopeptidase in L5 may be an important strategy used by *A. cantonensis* for cuticle synthesis and blood-brain barrier permeation.

Lysosome was the only pathway that was markedly enriched in the KEGG pathway analysis of the L5 and female adult transcriptomes. Lysosomes contain > 60 acidic lysosomal hydrolases belonging to different protein families. This enzymatic diversity makes it possible for lysosomes to participate in many important cellular processes, including protein secretion, macromolecular degradation, energy metabolism and pathogen defense (Saftig and Klumperman, 2009; Settembre *et al.*, 2012, 2013; Yu *et al.*, 2016). Alterations in the expression of genes coding for lysosomal enzymes will have a marked influence on lysosomal activity. The cuticle is a highly structured, collagenous extracellular matrix secreted by the hypodermis surrounding the worms body (Edgar *et al.*, 1982) and lysosomes may be involved in the metabolism of cuticlar collagen. Lysosomes also degrade and recycle a broad range of macromolecules as an important source of nutrients thereby providing an ingenious method for controlling and equilibrating anabolic and catabolic cellular processes (Luzio *et al.*, 2007). Thus, during the high-speed growth stage, lysosomes must adapt to meet the demand for new material and more energy during cuticle formation.

In this work, we compared the transcriptomes of *A. cantonensis* L5 and female adults. Our findings help to explain two main events that can impact parasite development and survival during this period. First, to meet the demands of rapid body growth, especially expansion of the body surface, L5 requires a large amount of material to sustain the formation of the stratum corneum and its associated structures. Based on the special role of lysosomes in autophagy and apoptosis, we speculate that these phenomena may play a certain role in altering the old and new cuticular tissues of these parasites. Secondly, metallopeptidases secreted by these parasites may be key molecules in allowing the parasite to break through the blood-brain barrier by degrading the extracellular matrix of host tissues. These findings offer novel insights into parasite development, survival and host-parasite interactions and provide a solid foundation for understanding how these genes participate in these processes.

## Supplementary Material

The following online material is available for this article:

Table S1 - Primers for qPCR used in this study (designed with Primer 3.0).

Table S2 - Summary of KEGG pathway annotation results for the P transcriptome.
