# Supplementary material for: Comparative transcriptomic analysis of two important life stages of Angiostrongylus cantonensis: fifth-stage larvae and female adults
Source: Genet Mol Biol. 2017 Apr-Jun;40(2):540–9. doi: 10.1590/1678-4685-GMB-2016-0274 (PMC5488468; doi:10.1590/1678-4685-GMB-2016-0274)
Supplement: Supplementary file 1 [file 1415-4757-gmb-40-02-0540-Suppl01.pdf]

Table S1 - Primers used for qPCR in this work (designed with Primer 3.0).

Unigenes	Primers	
c1248_g1	F	TGTGGACGTGGAAGCACTCG
	R	CATCAGAGTCGGCCATTGGA
c4480_g1	F	ATGTGATGCCCGATCCGACT
	R	GCGAGTCTCCAACACGACCA
c11627_g1	F	TGGCATTGGAGCTGAGGACA
	R	TGTGCCGTAACCGACGAGAA
c12074_g1	F	GGCGGCTCGTCAATAGTTGC
	R	TCAGGAAGGCTCGGCACTTT
c25913_g1	F	GCAGCGGAAGCAATGTCTGA
	R	GCTTGCAAACGTCCGCTTCT
c30842_g1	F	CGGTGGACTTCCAAGCCAAG
	R	AAATCGGCCCCTTCTTCACC
c33847_g1	F	CCGCAACGATACTCGGCTTC
	R	GGCGCCAATGTCAAGGAGTT
c35618_g1	F	CCGCCATAGTCGCTGTTGTG
	R	ACCCATAAGACGGCGCTTGA
c36992_g1	F	ACGCAAGTCGACGATGACCA
	R	CCCTTGGCTATCGCATGGAC
c38093_g1	F	CAGTGCTGCAGACCCCAGAG
	R	CGGCGTGTGATGTGAGATCC
c38662_g2	F	CAGACAACACTTGCCGCTGTG
	R	AGACCTCGTCTGCCGAATGG
c39588_g3	F	CATGGCGCAGAGGACTTGAA
	R	CCGCCACCACGAACATACTG
c62998_g2	F	GAGTGGGATTGGCGGAATGT
	R	TTCCAGCTCCAGAGCAGTCG
c63006_g1	F	AGGCGCTCCAAGAAATGCAC
	R	GCCAAGACATGGCAGGTTCA
c71175_g1	F	GCGATGCTTTCATGGCCTTC

Unigenes	Primers	
	R	CGGCGCCTATCAAGTGGTTC
Ac-β-actin	F	CCCAGAGCAGTCTTTCCTTCCA
	R	CCATAGGGTATTTCAGCGTTAG
