# Supplementary material for: Comparative transcriptomic analysis of two important life stages of Angiostrongylus cantonensis: fifth-stage larvae and female adults
Source: Genet Mol Biol. 2017 Apr-Jun;40(2):540–9. doi: 10.1590/1678-4685-GMB-2016-0274 (PMC5488468; doi:10.1590/1678-4685-GMB-2016-0274)
Supplement: Supplementary file 2 [file 1415-4757-gmb-40-02-0540-Suppl02.pdf]

Table S2 - Summary of the KEGG pathway annotation results for the P transcriptome.

Pathway Hierarchy1	Pathway Hierarchy2	KEGG Pathway	Pathway ID	Gene number
Metabolism	Amino acid metabolism	Lysine degradation	ko00310	225
Metabolism	Nucleotide metabolism	Purine metabolism	ko00230	122
Genetic Information Processing	Translation	Ribosome	ko03010	108
Genetic Information Processing	Transcription	Spliceosome	ko03040	100
Genetic Information Processing	Folding, sorting and degradation	Protein processing in endoplasmic reticulum	ko04141	99
Metabolism	Overview	Carbon metabolism	ko01200	92
Environmental Information Processing	Signal transduction	cAMP signaling pathway	ko04024	91
Cellular Processes	Cellular community	Focal adhesion	ko04510	90
Genetic Information Processing	Translation	RNA transport	ko03013	90
Metabolism	Energy metabolism	Oxidative phosphorylation	ko00190	89
Metabolism	Nucleotide metabolism	Pyrimidine metabolism	ko00240	83
Organismal Systems	Endocrine system	Insulin signaling pathway	ko04910	83
Organismal Systems	Endocrine system	Oxytocin signaling pathway	ko04921	83
Environmental Information Processing	Signal transduction	MAPK signaling pathway	ko04010	80
Cellular Processes	Cell motility	Regulation of actin cytoskeleton	ko04810	78

Pathway Hierarchy1	Pathway Hierarchy2	KEGG Pathway	Pathway ID	Gene number
Cellular Processes	Transport and catabolism	Phagosome	ko04145	78
Cellular Processes	Transport and catabolism	Endocytosis	ko04144	77
Cellular Processes	Transport and catabolism	Lysosome	ko04142	77
Environmental Information Processing	Signal transduction	cGMP-PKG signaling pathway	ko04022	77
Environmental Information Processing	Signal transduction	PI3K-Akt signaling pathway	ko04151	76
Cellular Processes	Cell growth and death	Cell cycle	ko04110	75
Cellular Processes	Cell growth and death	Oocyte meiosis	ko04114	74
Environmental Information Processing	Signal transduction	Rap1 signaling pathway	ko04015	74
Organismal Systems	Nervous system	Dopaminergic synapse	ko04728	71
Cellular Processes	Cellular community	Tight junction	ko04530	68
Genetic Information Processing	Translation	mRNA surveillance pathway	ko03015	68
Environmental Information Processing	Signal transduction	Hippo signaling pathway	ko04390	67
Organismal Systems	Circulatory system	Adrenergic signaling in cardiomyocytes	ko04261	65
Organismal Systems	Endocrine system	Thyroid hormone signaling pathway	ko04919	63
Cellular Processes	Transport and catabolism	Peroxisome	ko04146	62

Pathway Hierarchy1	Pathway Hierarchy2	KEGG Pathway	Pathway ID	Gene number
Organismal Systems	Circulatory system	Vascular smooth muscle contraction	ko04270	61
Environmental Information Processing	Signal transduction	Ras signaling pathway	ko04014	60
Genetic Information Processing	Translation	Ribosome biogenesis in eukaryotes	ko03008	60
Cellular Processes	Cell growth and death	Meiosis - yeast	ko04113	59
Environmental Information Processing	Signal transduction	AMPK signaling pathway	ko04152	59
Genetic Information Processing	Folding, sorting and degradation	Ubiquitin mediated proteolysis	ko04120	58
Organismal Systems	Immune system	Platelet activation	ko04611	58
Metabolism	Overview	Biosynthesis of amino acids	ko01230	55
Environmental Information Processing	Signal transduction	Wnt signaling pathway	ko04310	53
Organismal Systems	Nervous system	Neurotrophin signaling pathway	ko04722	53
Environmental Information Processing	Signal transduction	Calcium signaling pathway	ko04020	51
Organismal Systems	Endocrine system	Melanogenesis	ko04916	51
Environmental Information Processing	Signal transduction	FoxO signaling pathway	ko04068	50
Organismal Systems	Sensory system	Inflammatory mediator regulation of TRP channels	ko04750	50

Pathway Hierarchy1	Pathway Hierarchy2	KEGG Pathway	Pathway ID	Gene number
Metabolism	Carbohydrate metabolism	Glycolysis / Gluconeogenesis	ko00010	49
Organismal Systems	Immune system	Chemokine signaling pathway	ko04062	48
Cellular Processes	Cell growth and death	Cell cycle - yeast	ko04111	47
Cellular Processes	Cellular community	Gap junction	ko04540	46
Environmental Information Processing	Signal transduction	HIF-1 signaling pathway	ko04066	45
Genetic Information Processing	Folding, sorting and degradation	Proteasome	ko03050	45
Organismal Systems	Nervous system	GABAergic synapse	ko04727	45
Organismal Systems	Nervous system	Long-term potentiation	ko04720	45
Metabolism	Lipid metabolism	Glycerophospholipid metabolism	ko00564	44
Cellular Processes	Cellular community	Adherens junction	ko04520	43
Metabolism	Carbohydrate metabolism	Amino sugar and nucleotide sugar metabolism	ko00520	42
Organismal Systems	Endocrine system	Estrogen signaling pathway	ko04915	42
Organismal Systems	Immune system	Leukocyte transendothelial migration	ko04670	42
Organismal Systems	Nervous system	Glutamatergic synapse	ko04724	42
Metabolism	Carbohydrate metabolism	Pyruvate metabolism	ko00620	40
Organismal Systems	Nervous system	Serotonergic synapse	ko04726	40

Pathway Hierarchy1	Pathway Hierarchy2	KEGG Pathway	Pathway ID	Gene number
Organismal Systems	Nervous system	Synaptic vesicle cycle	ko04721	40
Environmental Information Processing	Signal transduction	Hippo signaling pathway -fly	ko04391	39
Genetic Information Processing	Folding, sorting and degradation	RNA degradation	ko03018	39
Metabolism	Carbohydrate metabolism	Citrate cycle (TCA cycle)	ko00020	39
Organismal Systems	Endocrine system	GnRH signaling pathway	ko04912	39
Organismal Systems	Nervous system	Cholinergic synapse	ko04725	39
Environmental Information Processing	Signal transduction	ErbB signaling pathway	ko04012	38
Organismal Systems	Digestive system	Pancreatic secretion	ko04972	37
Organismal Systems	Environmental adaptation	Circadian entrainment	ko04713	37
Organismal Systems	Nervous system	Retrograde endocannabinoid signaling	ko04723	37
Environmental Information Processing	Signaling molecules and interaction	Neuroactive ligand-receptor interaction	ko04080	36
Metabolism	Amino acid metabolism	Valine, leucine and isoleucine degradation	ko00280	36
Organismal Systems	Endocrine system	Progesterone-mediated oocyte maturation	ko04914	36
Genetic Information Processing	Translation	Aminoacyl-tRNA biosynthesis	ko00970	35
Organismal Systems	Excretory system	Vasopressin-regulated water reabsorption	ko04962	35

Pathway Hierarchy1	Pathway Hierarchy2	KEGG Pathway	Pathway ID	Gene number
Organismal Systems	Development	Axon guidance	ko04360	34
Organismal Systems	Digestive system	Salivary secretion	ko04970	34
Organismal Systems	Immune system	Fc gamma R-mediated phagocytosis	ko04666	34
Environmental Information Processing	Signal transduction	Phosphatidylinositol signaling system	ko04070	33
Genetic Information Processing	Transcription	RNA polymerase	ko03020	33
Metabolism	Overview	Fatty acid metabolism	ko01212	33
Metabolism	Amino acid metabolism	Arginine and proline metabolism	ko00330	32
Genetic Information Processing	Replication and repair	Nucleotide excision repair	ko03420	31
Organismal Systems	Digestive system	Bile secretion	ko04976	31
Organismal Systems	Immune system	T cell receptor signaling pathway	ko04660	31
Environmental Information Processing	Signal transduction	mTOR signaling pathway	ko04150	30
Metabolism	Glycan biosynthesis and metabolism	N-Glycan biosynthesis	ko00510	30
Organismal Systems	Endocrine system	PPAR signaling pathway	ko03320	30
Organismal Systems	Endocrine system	Thyroid hormone synthesis	ko04918	30
Organismal Systems	Sensory system	Phototransduction - fly	ko04745	30
Genetic Information Processing	Replication and repair	DNA replication	ko03030	28

Pathway Hierarchy1	Pathway Hierarchy2	KEGG Pathway	Pathway ID	Gene number
Genetic Information Processing	Transcription	Basal transcription factors	ko03022	28
Metabolism	Glycan biosynthesis and metabolism	Various types of N-glycan biosynthesis	ko00513	28
Metabolism	Lipid metabolism	Fatty acid degradation	ko00071	28
Environmental Information Processing	Membrane transport	ABC transporters	ko02010	27
Metabolism	Amino acid metabolism	Cysteine and methionine metabolism	ko00270	27
Metabolism	Carbohydrate metabolism	Fructose and mannose metabolism	ko00051	27
Organismal Systems	Circulatory system	Cardiac muscle contraction	ko04260	27
Organismal Systems	Digestive system	Gastric acid secretion	ko04971	27
Organismal Systems	Immune system	Antigen processing and presentation	ko04612	27
Metabolism	Carbohydrate metabolism	Starch and sucrose metabolism	ko00500	26
Metabolism	Metabolism of other amino acids	Glutathione metabolism	ko00480	26
Organismal Systems	Endocrine system	Insulin secretion	ko04911	26
Environmental Information Processing	Signal transduction	Notch signaling pathway	ko04330	25
Environmental Information Processing	Signal transduction	VEGF signaling pathway	ko04370	25
Metabolism	Amino acid metabolism	Alanine, aspartate and glutamate metabolism	ko00250	25

Pathway Hierarchy1	Pathway Hierarchy2	KEGG Pathway	Pathway ID	Gene number
Metabolism	Lipid metabolism	Glycerolipid metabolism	ko00561	25
Organismal Systems	Digestive system	Protein digestion and absorption	ko04974	25
Organismal Systems	Endocrine system	Adipocytokine signaling pathway	ko04920	25
Organismal Systems	Immune system	Fc epsilon RI signaling pathway	ko04664	25
Environmental Information Processing	Signal transduction	TGF-beta signaling pathway	ko04350	24
Metabolism	Carbohydrate metabolism	Inositol phosphate metabolism	ko00562	24
Organismal Systems	Endocrine system	Prolactin signaling pathway	ko04917	24
Organismal Systems	Nervous system	Long-term depression	ko04730	24
Metabolism	Energy metabolism	Carbon fixation in photosynthetic organisms	ko00710	23
Organismal Systems	Development	Osteoclast differentiation	ko04380	23
Environmental Information Processing	Signal transduction	TNF signaling pathway	ko04668	22
Metabolism	Amino acid metabolism	Tryptophan metabolism	ko00380	22
Organismal Systems	Immune system	Natural killer cell mediated cytotoxicity	ko04650	22
Genetic Information Processing	Folding, sorting and degradation	Protein export	ko03060	21
Metabolism	Amino acid metabolism	Glycine, serine and threonine metabolism	ko00260	21
Metabolism	Carbohydrate metabolism	Glyoxylate and dicarboxylate metabolism	ko00630	21

Pathway Hierarchy1	Pathway Hierarchy2	KEGG Pathway	Pathway ID	Gene number
Metabolism	Lipid metabolism	Sphingolipid metabolism	ko00600	21
Metabolism	Xenobiotics biodegradation and metabolism	Drug metabolism - other enzymes	ko00983	21
Organismal Systems	Immune system	B cell receptor signaling pathway	ko04662	21
Cellular Processes	Cell growth and death	p53 signaling pathway	ko04115	20
Environmental Information Processing	Signal transduction	Hedgehog signaling pathway	ko04340	20
Metabolism	Carbohydrate metabolism	Pentose and glucuronate interconversions	ko00040	20
Organismal Systems	Immune system	Toll-like receptor signaling pathway	ko04620	20
Metabolism	Carbohydrate metabolism	Galactose metabolism	ko00052	19
Metabolism	Carbohydrate metabolism	Propanoate metabolism	ko00640	19
Metabolism	Metabolism of other amino acids	[illegible]-Alanine metabolism	ko00410	19
Organismal Systems	Excretory system	Endocrine and other factor-regulated calcium reabsorption	ko04961	19
Metabolism	Carbohydrate metabolism	Pentose phosphate pathway	ko00030	18
Genetic Information Processing	Folding, sorting and degradation	SNARE interactions in vesicular transport	ko04130	17
Metabolism	Lipid metabolism	Ether lipid metabolism	ko00565	17
Organismal Systems	Digestive system	Mineral absorption	ko04978	17

Pathway Hierarchy1	Pathway Hierarchy2	KEGG Pathway	Pathway ID	Gene number
Environmental Information Processing	Signal transduction	Jak-STAT signaling pathway	ko04630	16
Metabolism	Carbohydrate metabolism	Butanoate metabolism	ko00650	16
Metabolism	Metabolism of terpenoids and polyketides	Terpenoid backbone biosynthesis	ko00900	16
Organismal Systems	Excretory system	Collecting duct acid secretion	ko04966	16
Genetic Information Processing	Replication and repair	Base excision repair	ko03410	15
Genetic Information Processing	Replication and repair	Fanconi anemia pathway	ko03460	15
Metabolism	Amino acid metabolism	Tyrosine metabolism	ko00350	15
Metabolism	Energy metabolism	Carbon fixation pathways in prokaryotes	ko00720	15
Metabolism	Lipid metabolism	Arachidonic acid metabolism	ko00590	15
Metabolism	Lipid metabolism	Fatty acid elongation	ko00062	15
Genetic Information Processing	Replication and repair	Mismatch repair	ko03430	14
Metabolism	Energy metabolism	Methane metabolism	ko00680	14
Metabolism	Metabolism of cofactors and vitamins	Pantothenate and CoA biosynthesis	ko00770	14
Metabolism	Metabolism of cofactors and vitamins	Retinol metabolism	ko00830	14
Metabolism	Overview	2-Oxocarboxylic acid metabolism	ko01210	14
Organismal Systems	Environmental adaptation	Plant-pathogen interaction	ko04626	14

Pathway Hierarchy1	Pathway Hierarchy2	KEGG Pathway	Pathway ID	Gene number
Organismal Systems	Excretory system	Proximal tubule bicarbonate reclamation	ko04964	14
Cellular Processes	Cell growth and death	Apoptosis	ko04210	13
Metabolism	Glycan biosynthesis and metabolism	Glycosaminoglycan biosynthesis - heparan sulfate / heparin	ko00534	13
Metabolism	Glycan biosynthesis and metabolism	Other glycan degradation	ko00511	13
Metabolism	Xenobiotics biodegradation and metabolism	Drug metabolism - cytochrome P450	ko00982	13
Organismal Systems	Development	Dorso-ventral axis formation	ko04320	13
Organismal Systems	Immune system	Cytosolic DNA-sensing pathway	ko04623	13
Organismal Systems	Sensory system	Olfactory transduction	ko04740	13
Metabolism	Amino acid metabolism	Histidine metabolism	ko00340	12
Metabolism	Carbohydrate metabolism	Ascorbate and aldarate metabolism	ko00053	12
Metabolism	Glycan biosynthesis and metabolism	Other types of O-glycan biosynthesis	ko00514	12
Metabolism	Lipid metabolism	Biosynthesis of unsaturated fatty acids	ko01040	12
Metabolism	Metabolism of cofactors and vitamins	Porphyrin and chlorophyll metabolism	ko00860	12
Organismal Systems	Excretory system	Aldosterone-regulated sodium reabsorption	ko04960	12
Organismal Systems	Sensory system	Phototransduction	ko04744	12
Cellular Processes	Transport and catabolism	Regulation of autophagy	ko04140	11

Pathway Hierarchy1	Pathway Hierarchy2	KEGG Pathway	Pathway ID	Gene number
Genetic Information Processing	Replication and repair	Homologous recombination	ko03440	11
Metabolism	Lipid metabolism	□-Linolenic acid metabolism	ko00592	11
Metabolism	Metabolism of cofactors and vitamins	One carbon pool by folate	ko00670	11
Metabolism	Metabolism of other amino acids	Selenocompound metabolism	ko00450	11
Metabolism	Xenobiotics biodegradation and metabolism	Metabolism of xenobiotics by cytochrome P450	ko00980	11
Organismal Systems	Digestive system	Carbohydrate digestion and absorption	ko04973	11
Environmental Information Processing	Signal transduction	MAPK signaling pathway - yeast	ko04011	10
Metabolism	Glycan biosynthesis and metabolism	Glycosaminoglycan degradation	ko00531	10
Metabolism	Glycan biosynthesis and metabolism	Glycosylphosphatidylinositol (GPI)-anchor biosynthesis	ko00563	10
Metabolism	Glycan biosynthesis and metabolism	Mucin type O-glycan biosynthesis	ko00512	10
Organismal Systems	Digestive system	Fat digestion and absorption	ko04975	10
Organismal Systems	Environmental adaptation	Circadian rhythm	ko04710	10
Environmental Information Processing	Signal transduction	NF-kappa B signaling pathway	ko04064	9
Environmental Information Processing	Signal transduction	Two-component system	ko02020	9

Pathway Hierarchy1	Pathway Hierarchy2	KEGG Pathway	Pathway ID	Gene number
Genetic Information Processing	Folding, sorting and degradation	Sulfur relay system	ko04122	9
Metabolism	Biosynthesis of other secondary metabolites	Isoquinoline alkaloid biosynthesis	ko00950	9
Metabolism	Energy metabolism	Nitrogen metabolism	ko00910	9
Metabolism	Energy metabolism	Sulfur metabolism	ko00920	9
Metabolism	Lipid metabolism	Fatty acid biosynthesis	ko00061	9
Metabolism	Lipid metabolism	Steroid hormone biosynthesis	ko00140	9
Metabolism	Metabolism of cofactors and vitamins	Nicotinate and nicotinamide metabolism	ko00760	9
Metabolism	Metabolism of cofactors and vitamins	Riboflavin metabolism	ko00740	9
Organismal Systems	Immune system	RIG-I-like receptor signaling pathway	ko04622	9
Environmental Information Processing	Signaling molecules and interaction	ECM-receptor interaction	ko04512	8
Metabolism	Amino acid metabolism	Phenylalanine metabolism	ko00360	8
Metabolism	Biosynthesis of other secondary metabolites	Streptomycin biosynthesis	ko00521	8
Metabolism	Metabolism of cofactors and vitamins	Folate biosynthesis	ko00790	8
Organismal Systems	Digestive system	Vitamin digestion and absorption	ko04977	8
Organismal Systems	Immune system	NOD-like receptor signaling pathway	ko04621	8
Organismal Systems	Sensory system	Taste transduction	ko04742	8

Pathway Hierarchy1	Pathway Hierarchy2	KEGG Pathway	Pathway ID	Gene number
Environmental Information Processing	Signal transduction	MAPK signaling pathway - fly	ko04013	7
Metabolism	Lipid metabolism	Linoleic acid metabolism	ko00591	7
Metabolism	Xenobiotics biodegradation and metabolism	Aminobenzoate degradation	ko00627	7
Organismal Systems	Endocrine system	Ovarian steroidogenesis	ko04913	7
Metabolism	Glycan biosynthesis and metabolism	Glycosaminoglycan biosynthesis - chondroitin sulfate / dermatan sulfate	ko00532	6
Metabolism	Xenobiotics biodegradation and metabolism	Caprolactam degradation	ko00930	6
Environmental Information Processing	Signaling molecules and interaction	Cell adhesion molecules (CAMs)	ko04514	5
Environmental Information Processing	Signaling molecules and interaction	Cytokine-cytokine receptor interaction	ko04060	5
Genetic Information Processing	Replication and repair	Non-homologous end-joining	ko03450	5
Metabolism	Glycan biosynthesis and metabolism	Glycosphingolipid biosynthesis - lacto and neolacto series	ko00601	5
Metabolism	Lipid metabolism	Primary bile acid biosynthesis	ko00120	5
Metabolism	Metabolism of cofactors and vitamins	Ubiquinone and other terpenoid-quinone biosynthesis	ko00130	5
Metabolism	Xenobiotics biodegradation and metabolism	Benzoate degradation	ko00362	5
Organismal Systems	Environmental adaptation	Circadian rhythm - fly	ko04711	5

Pathway Hierarchy1	Pathway Hierarchy2	KEGG Pathway	Pathway ID	Gene number
Metabolism	Amino acid metabolism	Phenylalanine, tyrosine and tryptophan biosynthesis	ko00400	4
Metabolism	Amino acid metabolism	Valine, leucine and isoleucine biosynthesis	ko00290	4
Metabolism	Biosynthesis of other secondary metabolites	Betalain biosynthesis	ko00965	4
Metabolism	Biosynthesis of other secondary metabolites	Tropane, piperidine and pyridine alkaloid biosynthesis	ko00960	4
Metabolism	Glycan biosynthesis and metabolism	Glycosaminoglycan biosynthesis - keratan sulfate	ko00533	4
Metabolism	Glycan biosynthesis and metabolism	Glycosphingolipid biosynthesis - globo series	ko00603	4
Metabolism	Lipid metabolism	Steroid biosynthesis	ko00100	4
Metabolism	Lipid metabolism	Synthesis and degradation of ketone bodies	ko00072	4
Metabolism	Metabolism of other amino acids	Taurine and hypotaurine metabolism	ko00430	4
Metabolism	Metabolism of terpenoids and polyketides	Geraniol degradation	ko00281	4
Metabolism	Xenobiotics biodegradation and metabolism	Styrene degradation	ko00643	4
Organismal Systems	Endocrine system	Renin-angiotensin system	ko04614	4
Cellular Processes	Cell growth and death	Cell cycle - Caulobacter	ko04112	3
Metabolism	Biosynthesis of other secondary metabolites	Butirosin and neomycin biosynthesis	ko00524	3
Metabolism	Glycan biosynthesis and metabolism	Glycosphingolipid biosynthesis - ganglio series	ko00604	3

Pathway Hierarchy1	Pathway Hierarchy2	KEGG Pathway	Pathway ID	Gene number
Metabolism	Metabolism of other amino acids	Cyanoamino acid metabolism	ko00460	3
Metabolism	Metabolism of other amino acids	D-Glutamine and D-glutamate metabolism	ko00471	3
Metabolism	Metabolism of other amino acids	Phosphonate and phosphinate metabolism	ko00440	3
Metabolism	Metabolism of terpenoids and polyketides	Limonene and pinene degradation	ko00903	3
Environmental Information Processing	Membrane transport	Bacterial secretion system	ko03070	2
Metabolism	Biosynthesis of other secondary metabolites	Indole alkaloid biosynthesis	ko00901	2
Metabolism	Energy metabolism	Photosynthesis - antenna proteins	ko00196	2
Metabolism	Metabolism of cofactors and vitamins	Lipoic acid metabolism	ko00785	2
Metabolism	Metabolism of cofactors and vitamins	Vitamin B6 metabolism	ko00750	2
Metabolism	Metabolism of terpenoids and polyketides	Polyketide sugar unit biosynthesis	ko00523	2
Metabolism	Overview	Degradation of aromatic compounds	ko01220	2
Metabolism	Xenobiotics biodegradation and metabolism	Bisphenol degradation	ko00363	2
Metabolism	Xenobiotics biodegradation and metabolism	Chloroalkane and chloroalkene degradation	ko00625	2
Organismal Systems	Environmental adaptation	Circadian rhythm - plant	ko04712	2
Organismal Systems	Immune system	Hematopoietic cell lineage	ko04640	2
Metabolism	Amino acid metabolism	Lysine biosynthesis	ko00300	1

Pathway Hierarchy1	Pathway Hierarchy2	KEGG Pathway	Pathway ID	Gene number
Metabolism	Biosynthesis of other secondary metabolites	Aflatoxin biosynthesis	ko00254	1
Metabolism	Biosynthesis of other secondary metabolites	Caffeine metabolism	ko00232	1
Metabolism	Biosynthesis of other secondary metabolites	Flavone and flavonol biosynthesis	ko00944	1
Metabolism	Biosynthesis of other secondary metabolites	Novobiocin biosynthesis	ko00401	1
Metabolism	Biosynthesis of other secondary metabolites	Stilbenoid, diarylheptanoid and gingerol biosynthesis	ko00945	1
Metabolism	Energy metabolism	Photosynthesis	ko00195	1
Metabolism	Lipid metabolism	Cutin, suberine and wax biosynthesis	ko00073	1
Metabolism	Metabolism of cofactors and vitamins	Biotin metabolism	ko00780	1
Metabolism	Metabolism of cofactors and vitamins	Thiamine metabolism	ko00730	1
Metabolism	Metabolism of terpenoids and polyketides	Biosynthesis of ansamycins	ko01051	1
Metabolism	Metabolism of terpenoids and polyketides	Biosynthesis of vancomycin group antibiotics	ko01055	1
Metabolism	Metabolism of terpenoids and polyketides	Insect hormone biosynthesis	ko00981	1
Metabolism	Metabolism of terpenoids and polyketides	Zeatin biosynthesis	ko00908	1
Metabolism	Xenobiotics biodegradation and metabolism	Polycyclic aromatic hydrocarbon degradation	ko00624	1
Organismal Systems	Immune system	Complement and coagulation cascades	ko04610	1
